# Ivabradine improves win ratios of heart failure outcomes in patients with reduced ejection fraction – insights from the SHIFT trial

**DOI:** 10.1002/ejhf.3648

**Published:** 2025-03-30

**Authors:** Amr Abdin, Saarraaken Kulenthiran, Michel Komajda, Jeffrey S. Borer, Ian Ford, Luigi Tavazzi, Cécile Batailler, Karl Swedberg, Michael Böhm, on behalf of the SHIFT Investigators

**Affiliations:** ^1^ Department of Internal Medicine III, Cardiology, Angiology, Intensive Care Medicine Saarland University Medical Center Homburg Germany; ^2^ Department of Cardiology Hospital Saint Joseph Paris France; ^3^ The Howard Gilman Institute for Heart Valve Diseases and Schiavone Institute for Cardiovascular Translational Research State University of New York Downstate Health Sciences University Brooklyn and New York NY USA; ^4^ Robertson Centre for Biostatistics University of Glasgow Glasgow UK; ^5^ Maria Cecilia Hospital, GVM Care & Research Cotignola Italy; ^6^ Institut de Recherches Internationales Servier Suresnes France; ^7^ Department of Molecular and Clinical Medicine University of Gothenburg Gothenburg Sweden

Resting heart rate is a strong predictor of cardiovascular (CV) mortality and morbidity in patients with heart failure (HF).^1^ The results of the SHIFT trial showed that heart rate reduction with ivabradine significantly reduced adverse clinical outcomes in a population with symptomatic HF and heart rates of 70 bpm or more.^2^, ^3^ In CV outcome trials, treatment efficacy is often assessed with a composite endpoint that includes both fatal and non‐fatal events, using a Cox proportional hazards model focused on the time to the first event. This method has limitations, such as giving equal statistical weight to each component, regardless of its clinical significance.^4^ This is especially relevant as recent HF composites may include milder, non‐hospitalization events. Furthermore, the model overlooks fatal events that occur after non‐fatal ones and ignores recurrent non‐fatal episodes, which may lower the treatment's perceived impact by focusing only on the first event. Alternatively, the win ratio (WR) approach uses a composite outcome that aligns with clinical priorities and patient preferences.^5^ It enables a hierarchical structure based on the clinical importance of each component and can include recurrent events along with continuous or categorical measures, like patient‐reported outcomes or biomarkers.^6^ These advantages have recently drawn significant attention to the WR method. To understand how the WR compares to conventional time‐to‐first‐event and total events analyses, we conducted this study to evaluate WR alongside hazard ratios (HRs) within the SHIFT trial.

The SHIFT trial enrolled 6505 patients with left ventricular ejection fraction (LVEF) ≤35% and a resting heart rate ≥70 bpm. Patients were randomized to receive ivabradine or placebo in addition to guideline‐based standard care.^2^ The starting dose was 5 mg ivabradine twice daily; doses were adjusted upward or downward (2.5, 5, or 7.5 mg twice daily) at every visit according to heart rate and tolerability. Ivabradine reduced CV deaths or HF hospitalizations (HFH) (the study primary endpoint) by 18% (*p* < 0.0001), primarily driven by reduced hospital admissions for worsening HF (HR 0.74, 95% confidence interval [CI] 0.66–0.83; *p* < 0.0001).

In the present analysis, the clinical benefit was assessed using the WR with a hierarchical endpoint of CV death and number of HFH. For this analysis, we used both matched and unmatched methods.^5^, ^7^ In the matched pairs approach, patients receiving the new treatment were paired with those on standard treatment based on individual risk scores. In SHIFT, these scores were calculated using a Cox model adjusted for key prognostic factors: beta‐blocker use, New York Heart Association class, LVEF, age, ischaemia, systolic blood pressure, and baseline creatinine clearance, as detailed in the statistical plan. This produced one risk score per patient. There were 3241 patients on ivabradine and 3264 on placebo, so we removed 23 from the placebo group to equalize the groups. Patients were ranked by risk score, and each ivabradine patient was paired with a placebo patient of the same rank, resulting in 3231 pairs, excluding those with missing data. We then applied methods to determine winners and calculated the WR.

The unmatched approach involved comparing each new treatment patient with each standard treatment patient, recording the ‘winner’ based on event outcomes. Comparisons proceeded by event tiers, starting with CV death during a shared follow‐up period (tier 1). If that tier was settled, we moved to tier 2, comparing the number of HFH. If both patients had the same number of hospitalizations, we compared the time to first hospitalization. The same approach was used for all‐cause mortality and number of HFH. To account for different follow‐up periods, the win rate typically includes time‐to‐event data, so it captures whether a treatment is more effective at preventing or delaying adverse outcomes over the entire observation period. If some patients are followed for a shorter period, their ‘win’ status may be less certain, but this is adjusted for by including censoring, which means it is not necessary that all patients are followed for exactly the same length of time.

**Figure 1:**
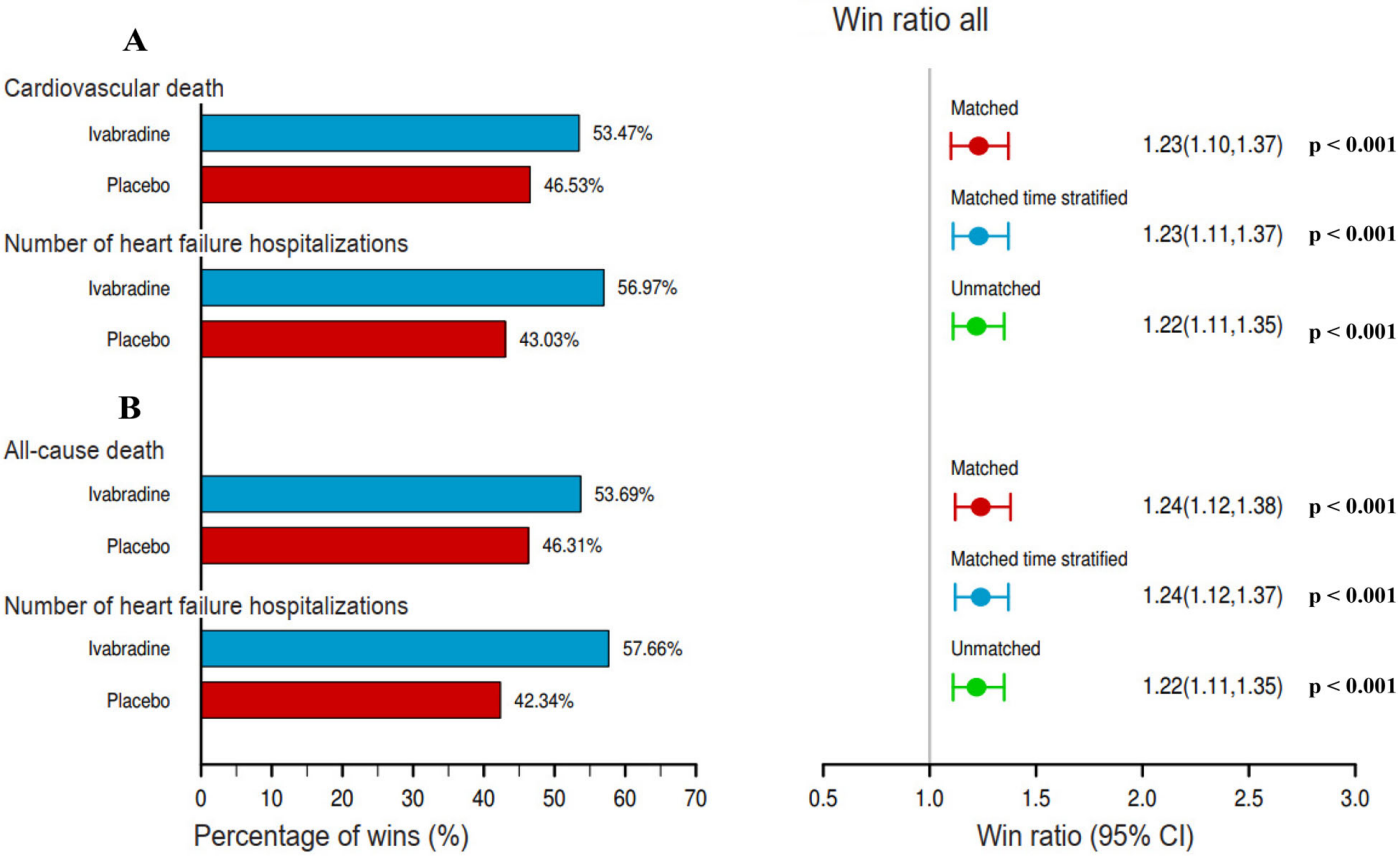
Win ratios for (*A*) the primary composite endpoint (cardiovascular death or number of heart failure hospitalizations) and (*B*) the composite endpoint (all‐cause death or number of heart failure hospitalizations) using matched and unmatched methods. CI, confidence interval.

Ivabradine showed a statistically significant benefit over placebo for the primary endpoint, with a WR of 1.23 (95% CI 1.10–1.37; *p* < 0.001), consistent across both CV deaths (53.47% vs. 46.53%, *p* < 0.05) and number of HFH (56.97% vs. 43.03%, *p* < 0.001) (*Figure* *1A*). No difference was observed between the matched and unmatched analyses, with WRs of 1.23 (95% CI 1.11–1.37; *p* < 0.001) and 1.22 (95% CI 1.11–1.35; *p* < 0.001), respectively.

Similarly, for the combined endpoint of all‐cause mortality and total HFH, ivabradine demonstrated a significant benefit with a WR of 1.24 (95% CI 1.12–1.38; *p* < 0.001), consistent across all‐cause deaths (53.69% vs. 46.31%, *p* < 0.001) and HFH (57.66% vs. 42.34%, *p* < 0.001) (*Figure* *1B*).

In the SHIFT trial, ivabradine did not significantly reduce CV death (HR 0.91; 95% CI 0.80–1.03, *p* = 0.12) but did reduce HFH (HR 0.89; 0.82–0.96, *p* = 0.003).^2^ This led to an ‘overestimation’ of the effect in the Cox model, which ignored CV deaths occurring after HFH, unlike the WR, which accounted for all CV deaths. In this analysis, the WR for the composite of these events produced a lower *p*‐value than the HR, indicating that WR may be more clinically meaningful by prioritizing fatal events. The WR over time provides insights into the treatment impact on each component of the outcome (fatal and non‐fatal events), even when they differ in timing or direction.^8^

Like any test, both the Cox and WR methods have limitations. There are currently no established methods to determine sample size and power for the WR, nor is there a standard approach to adjust for covariates—though this is less critical in large randomized trials where baseline variables are generally balanced. The WR is designed to maximize statistical power with a smaller sample size. While this approach can be advantageous, it may mean that the method prioritizes certain outcomes over a broader, holistic understanding of treatment effects across the entire population.^9^ Clinician familiarity with the WR remains limited, though it may increase with more widespread use. Additionally, the WR does not account for precise time to event, only noting whether an event occurred before or after the same event in the patient pair. In addition, the WR method can struggle with censoring (i.e. when patients are lost to follow‐up or do not experience the event within the study period). Our analysis also has limitations, as we did not explore other potential applications of WR, such as continuous outcomes.

These analyses offered a detailed set of win statistics, showcasing their flexibility in evaluating treatment effects. Ivabradine consistently showed clinical benefits across various statistical methods. With recent advancements in win statistics and visualization tools, these approaches provide an alternative to traditional clinical trial analysis methods.

### Funding

Michael Böhm is supported by the Deutsche Forschungsgemeinschaft (German Research Foundation; TTR 219, project number 322900939).

**Conflict of interest**: M.K. reports consulting, speaker activities, or member of clinical trial committees for Novartis Servier Bohringer Ingelheim and Bayer. I.F. reports grants from Kidney Research UK, during the conduct of the study; grants from Vifor Pharma, Pharmacosmos, outside the submitted work. L.T. reports honoraria from Servier as trial committee member. C.B. is an employee of Servier France. K.S. reports honoraria from AstraZeneca, Boehringer, Novartis, Pfizer. M.B. reports personal fees from Abbott, Amgen, AstraZeneca, Bayer, Boehringer Ingelheim, Cytokinetics, Medtronic, Novartis, ReCor Servier and Vifor. All other authors have nothing to disclose.
